# 

**Published:** 1850-07

**Authors:** 


### CONTENTS OF VOLUME III.

Address of Dr. Goddard, before M. V. A. D. S., ........... 1
" " J. Taft, M. D., " " on Extraction of Teeth, ........... 15
Address of Dr. Leslie, on Mettallurgy, ........... 21
" Valedictory, to Graduating Class, Ohio C. D. S.—by Geo. Mendenhall, M. D., ........... 105
Advice on management of Teeth, ........... 87
Address on filing Teeth—by Dr. Curtiss, ........... 126
Application of Tortoise-Shell, ........... 190
Annual Meeting Am. So. Dental Surgeons, ........... 192
Case of third Dentition, ........... 75
Charleston Medical and Surgical Review, Ed., ........... 103
Contributions, Ed., ........... 156
Case of Neuralgia, ........... 179
Dental Notices, ........... 70
Disease of the Gums, ........... 77
Death from Chloroform, ........... 100
Dental Removals, Ed., ........... 103
Dental Retiracy, Ed., ........... 102
Dental Register, Ed., ........... 204
Dunlevy's Foil, Ed., ........... 206
Evans' New Amalgam, ........... 93
Exostosis, ........... 104
Errata, ........... 104
Fifth Annual Meeting M. V. A. D. D., ........... 37
Facial Neuralgia and Tic Douloureux, &c.—by Dr. S. P. Hullihen, ........... 66
Grinding Apparatus, ........... 74
Glimpses of Ancient Physic and Physicians, ........... 149
Harris' Principles and Practice of Dental Surgery, ........... 205
Insanity in the United States, ........... 102
Important Discovery in Dentistry, ........... 102
Inaugural Dissertation on Dental Decay, ........... 118
Lateral Cavity, Plates, Ed., ........... 155

Medical Notice, .......... 47
Moulding, .......... 76
Metallic Castings, .......... 96
Medical Testimony in Webster's Trial, .......... 147
Memoir of Dr. Nasmyth, .......... 166
Mississippi Asssociation of Dental Surgeons, .......... 205
New York Dental Recorder, Ed., .......... 47
Notice of New Book, Ed., .......... 103
Neuralgia vs. Toothache, .......... 188
Notice of Dr. Hullihen's Valedictory Address, Ed., .......... 200
Our next Number, Ed., .......... 48
Obituary, Ed., .......... 49 and 206
Odontalgia—by Drs. Hullihen and Robinson, .......... 79
Ohio College Dental Surgeons, Ed., .......... 156
Pivot Teeth—by A. Berry, D. D. S., .......... 46
Postponement Tenth Annual Meeting Am. S. D. S., .......... 46
Proceedings of Pennsylvania Society Dental Surgeons, .......... 83
" " Society Dental Surgeons N. Y., .......... 91
Physick's Forceps—A. Berry, D, D. S., .......... 128
Prof. Webster's Conviction, .......... 148
Partial Sets of Teeth—W. H. King, .......... 158
Preternatural Growth of the Gums—Dr. Taft, .......... 176
Pennsylvania Society Dental Surgeons, Ed., .......... 203
Quack Extortions, .......... 99
Report of the Committee of Penn. So. D. S., .......... 196
Report on Caries, &c.—Dr. B. T. Whitney, .......... 53
St. Louis Probe, Ed., .......... 156
Snuff-taking, &c.—R. A. Kinloch, M. D., .......... 181
Teeth Discolored by Arsenious Acid, .......... 176
Transplantation of Teeth—by James Gardette, D. D. S., .......... 130
Treatment of Pulp, &c.—J. D. White, D. D. S., .......... 138
Use of Clasps—P. H. Austen, M. D., .......... 143

